# 2-[(*E*)-(1*H*-Pyrrol-2-ylmethyl­idene)hydrazinyl]pyridine monohydrate

**DOI:** 10.1107/S1600536809050296

**Published:** 2009-11-28

**Authors:** Geraldo M. de Lima, Edward R. T. Tiekink, James L. Wardell, Solange M. S. V. Wardell

**Affiliations:** a Departamento de Quimica, ICEx, Universidade Federal de Minas Gerais, 31270-901 Belo Horizonte, MG, Brazil; b Department of Chemistry, University of Malaya, 50603 Kuala Lumpur, Malaysia; c Centro de Desenvolvimento Tecnológico em Saúde (CDTS), Fundação Oswaldo Cruz (FIOCRUZ), Casa Amarela, Campus de Manguinhos, Av. Brasil 4365, 21040-900 Rio de Janeiro, RJ, Brazil; d CHEMSOL, 1 Harcourt Road, Aberdeen AB15 5NY, Scotland

## Abstract

The title hydrate, C_10_H_10_N_4_·H_2_O, shows a small twist in the hydro­zone derivative, the dihedral angle between the pyridine and pyrrole rings being 11.08 (12)°. The pyridine and pyrrole N atoms lie to the same side of the mol­ecule being sustained in place by hydrogen-bonding inter­actions with the water mol­ecule. Further inter­molecular O—H⋯N and N—H⋯O hydrogen bonding leads to the formation of supra­molecular arrays in the *ab* plane.

## Related literature

For related structures of hydro­zone derivatives, see: Baddeley *et al.* (2009 ▶); Ferguson *et al.* (2005 ▶); Wardell, Low & Glidewell (2007 ▶); Wardell, Skakle, Low & Glidewell (2007 ▶). For additional structural analaysis, see: Spek (2003 ▶).
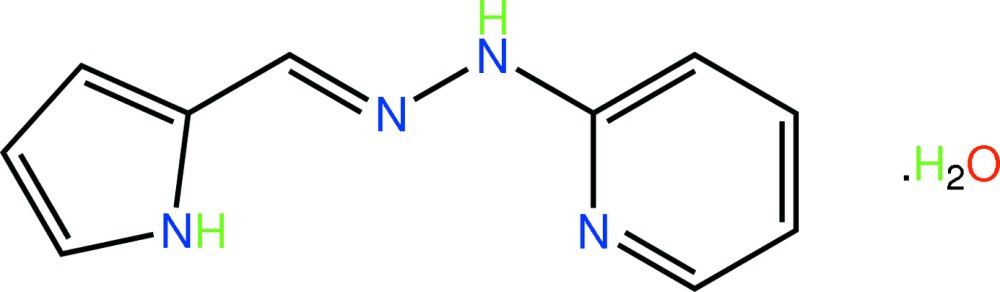


## Experimental

### 

#### Crystal data

C_10_H_10_N_4_·H_2_O
*M* *_r_* = 204.24
Monoclinic, 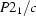

*a* = 5.6479 (3) Å
*b* = 7.4383 (4) Å
*c* = 24.4233 (11) Å
β = 103.300 (3)°
*V* = 998.52 (9) Å^3^
*Z* = 4
Mo *K*α radiation
μ = 0.09 mm^−1^
*T* = 120 K
0.24 × 0.22 × 0.04 mm

#### Data collection

Nonius KappaCCD area-detector diffractometer
Absorption correction: multi-scan (*SADABS*; Sheldrick, 2007 ▶) *T*_min_ = 0.670, *T*_max_ = 0.746
3068 measured reflections
1680 independent reflections
1636 reflections with *I* > 2σ(*I*)
*R*_int_ = 0.027

#### Refinement

*R*[*F* ^2^ > 2σ(*F* ^2^)] = 0.040
*wR*(*F* ^2^) = 0.109
*S* = 1.07
1680 reflections
149 parameters
4 restraints
H atoms treated by a mixture of independent and constrained refinement
Δρ_max_ = 0.19 e Å^−3^
Δρ_min_ = −0.24 e Å^−3^

### 

Data collection: *COLLECT* (Hooft, 1998 ▶); cell refinement: *DENZO* (Otwinowski & Minor, 1997 ▶) and *COLLECT* data reduction: *DENZO* nd *COLLECT*; program(s) used to solve structure: *SHELXS97* (Sheldrick, 2008 ▶); program(s) used to refine structure: *SHELXL97* (Sheldrick, 2008 ▶); molecular graphics: *DIAMOND* (Brandenburg, 2006 ▶); software used to prepare material for publication: *publCIF* (Westrip, 2009 ▶).

## Supplementary Material

Crystal structure: contains datablocks global, I. DOI: 10.1107/S1600536809050296/hg2608sup1.cif

Structure factors: contains datablocks I. DOI: 10.1107/S1600536809050296/hg2608Isup2.hkl

Additional supplementary materials:  crystallographic information; 3D view; checkCIF report

## Figures and Tables

**Table 1:** Hydrogen-bond geometry (Å, °)

*D*—H⋯*A*	*D*—H	H⋯*A*	*D*⋯*A*	*D*—H⋯*A*
O1w—H1w⋯N1	0.84 (1)	2.04 (1)	2.870 (2)	170 (3)
O1w—H2w⋯N3^i^	0.84 (1)	2.08 (1)	2.899 (3)	166 (3)
N2—H2n⋯O1w^ii^	0.89 (1)	2.09 (1)	2.959 (3)	166 (2)
N4—H4n⋯O1w	0.88 (1)	1.97 (1)	2.831 (2)	165 (2)

Symmetry codes: (i) 
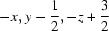
; (ii) 
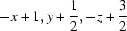
.

**Fig. 1.**
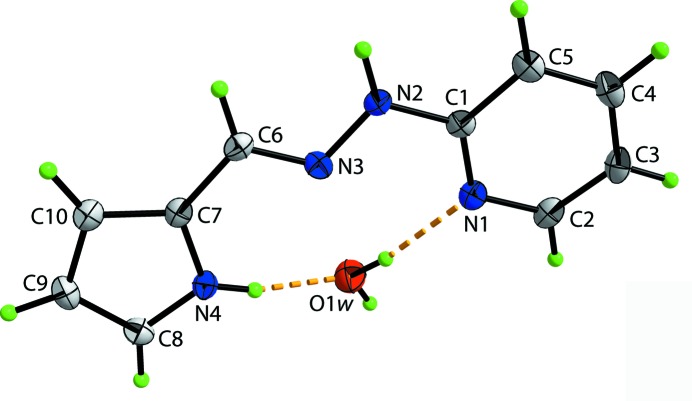
The molecular structure of (I) showing the atom-labelling scheme and displacement ellipsoids at the 50% probability level. The O—H···N and N—H···O hydrogen bonds are shown as orange dashed lines.

**Fig. 2.**
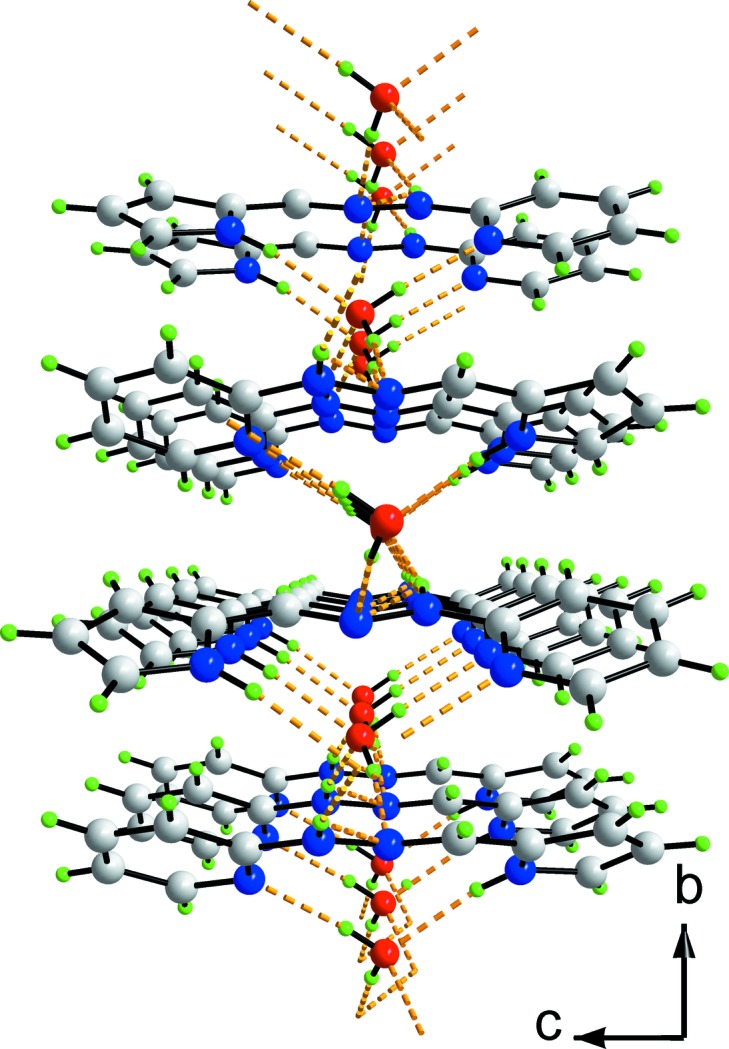
A side-on view of a supramolecular layer in (I) showing O–H···N and N–H···O hydrogen bonding between the molecules (orange dashed lines). Colour code: O, red; N, blue; C, grey; and H, green.

**Fig. 3.**
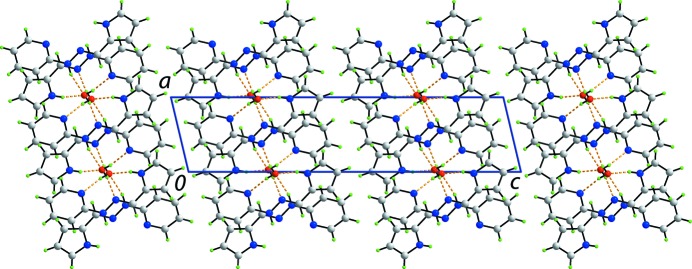
A view in projection down the b axis showing the stacking of layers along the c axis in (I). The O–H···N and N–H···O hydrogen bonding is shown as orange dashed lines. Colour code: O, red; N, blue; C, grey; and H, green.

## supplementary crystallographic information

### Comment

In continuation of studies into the supramolecular arrangements of hydrazones (Baddeley *et al.*, 2009; Wardell, Skakle *et al.*, 2007; Wardell, Low *et al.*, 2007; Ferguson *et al.*, 2005), we now report the structure of the title hydrate, (I).

The molecule of (I), Fig. 1, is non-planar owing to a twist about the C6–C7 bond as seen in the C3–C6–C7–N4 torsion angle of -11.2 (3) °. The dihedral angle between the pyrrole and pyridine rings is 11.08 (12) °. The conformation about the C6═N3 bond is E and the pyrrole- and pyridine-N atoms are *syn*. This arrangement is stabilized by pyrrole-NH···O_water_ and water-OH···N_pyridine_ hydrogen bonds, Fig. 1 and Table 1.

The water molecule also plays a pivotal role in stabilizing the crystal structure, forming additional donor and acceptor interactions to link three distinct molecules. The molecules stack into columns aligned along the *a* axis and alternate between organic and water molecules along the *b* axis, Fig. 2 and Table 1. The resultant layers stack along the *c* axis, Fig. 3.

### Experimental

A solution of 2-hydrazinopyridine (0.330 g, 3 mmol) in MeOH (15 ml) was added to a solution of 2-pyrrolcarboxaldehyde (0.270 g, 3 mmol) in MeOH (10 ml). The reaction mixture was refluxed for 20 min, and maintained at room temperature. The crystals which slowly formed were collected and recrystallized twice from MeOH. *M*. pt. 449 - 452 K. IR(KBr, cm^-1^): ν 1603(C=N). Anal. Found, C, 59.13; H, 5.81; N, 27.71. Calc. for C_10_H_10_N_4_.H_2_O: C, 58.82; H, 5.92; N, 27.43%

### Refinement

The C-bound H atoms were geometrically placed (C–H = 0.95 Å) and refined as riding with *U*_iso_(H) = 1.2*U*_eq_(C). The O– and N-bound H atoms were located from a difference map and included in their idealized positions with O–H = 0.84±0.01 and N–H = 0.88±0.01 Å, and with *U*_iso_(H) = n*U*_eq_(O, N); n = 1.5 for O and n = 1.2 for N. After analysis with *PLATON* (Spek, 2003), the structure was refined as a twin, with the twin component related by -1 0 0, 0 - 1 0, 2 0 1, and with a fractional contribution of 0.4418 (23).

### Crystal data

**Table:**

C_10_H_10_N_4_·H_2_O	*F*(000) = 432
*M**_r_* = 204.24	*D*_x_ = 1.359 Mg m^−^^3^
Monoclinic, *P*2_1_/*c*	Mo *K*α radiation, λ = 0.71073 Å
Hall symbol: -P 2ybc	Cell parameters from 18744 reflections
*a* = 5.6479 (3) Å	θ = 2.9–27.5°
*b* = 7.4383 (4) Å	µ = 0.09 mm^−^^1^
*c* = 24.4233 (11) Å	*T* = 120 K
β = 103.300 (3)°	Block, colourless
*V* = 998.52 (9) Å^3^	0.24 × 0.22 × 0.04 mm
*Z* = 4	

### Data collection

**Table:**

Nonius KappaCCD area-detector diffractometer	1680 independent reflections
Radiation source: Enraf Nonius FR591 rotating anode	1636 reflections with *I* > 2σ(*I*)
10 cm confocal mirrors	*R*_int_ = 0.027
Detector resolution: 9.091 pixels mm^-1^	θ_max_ = 25.0°, θ_min_ = 3.2°
φ and ω scans	*h* = −6→6
Absorption correction: multi-scan (*SADABS*; Sheldrick, 2007)	*k* = −8→8
*T*_min_ = 0.670, *T*_max_ = 0.746	*l* = −28→28
3068 measured reflections	

### Refinement

**Table:**

Refinement on *F*^2^	Primary atom site location: structure-invariant direct methods
Least-squares matrix: full	Secondary atom site location: difference Fourier map
*R*[*F*^2^ > 2σ(*F*^2^)] = 0.040	Hydrogen site location: inferred from neighbouring sites
*wR*(*F*^2^) = 0.109	H atoms treated by a mixture of independent and constrained refinement
*S* = 1.07	*w* = 1/[σ^2^(*F*_o_^2^) + (0.0651*P*)^2^ + 0.3833*P*] where *P* = (*F*_o_^2^ + 2*F*_c_^2^)/3
1680 reflections	(Δ/σ)_max_ < 0.001
149 parameters	Δρ_max_ = 0.19 e Å^−^^3^
4 restraints	Δρ_min_ = −0.24 e Å^−^^3^

### Special details

**Table:**

Geometry. All s.u.'s (except the s.u. in the dihedral angle between two l.s. planes) are estimated using the full covariance matrix. The cell s.u.'s are taken into account individually in the estimation of s.u.'s in distances, angles and torsion angles; correlations between s.u.'s in cell parameters are only used when they are defined by crystal symmetry. An approximate (isotropic) treatment of cell s.u.'s is used for estimating s.u.'s involving l.s. planes.
Refinement. Refinement of *F*^2^ against ALL reflections. The weighted *R*-factor *wR* and goodness of fit *S* are based on *F*^2^, conventional *R*-factors *R* are based on *F*, with *F* set to zero for negative *F*^2^. The threshold expression of *F*^2^ > 2σ(*F*^2^) is used only for calculating *R*-factors(gt) *etc*. and is not relevant to the choice of reflections for refinement. *R*-factors based on *F*^2^ are statistically about twice as large as those based on *F*, and *R*- factors based on ALL data will be even larger.

### Fractional atomic coordinates and isotropic or equivalent isotropic displacement parameters (Å^2^)

**Table:**

	*x*	*y*	*z*	*U*_iso_*/*U*_eq_	
O1W	0.0268 (3)	0.5726 (2)	0.74096 (6)	0.0234 (4)	
H1W	0.088 (5)	0.635 (3)	0.7695 (7)	0.035*	
H2W	−0.071 (4)	0.501 (3)	0.7507 (11)	0.035*	
N1	0.2897 (3)	0.7646 (2)	0.83791 (7)	0.0221 (4)	
N2	0.5539 (4)	0.8844 (2)	0.78669 (8)	0.0218 (4)	
H2N	0.694 (3)	0.935 (3)	0.7844 (10)	0.026*	
N3	0.3760 (4)	0.8612 (2)	0.73892 (7)	0.0205 (4)	
N4	0.0207 (4)	0.7784 (2)	0.64316 (7)	0.0205 (4)	
H4N	0.000 (5)	0.726 (3)	0.6740 (6)	0.025*	
C1	0.4955 (4)	0.8541 (3)	0.83784 (8)	0.0193 (4)	
C2	0.2360 (4)	0.7344 (3)	0.88823 (8)	0.0238 (5)	
H2	0.0904	0.6710	0.8886	0.029*	
C3	0.3790 (5)	0.7898 (3)	0.93898 (9)	0.0257 (5)	
H3	0.3346	0.7654	0.9735	0.031*	
C4	0.5912 (5)	0.8829 (3)	0.93755 (9)	0.0250 (5)	
H4	0.6942	0.9245	0.9716	0.030*	
C5	0.6526 (4)	0.9152 (3)	0.88711 (9)	0.0229 (5)	
H5	0.7981	0.9774	0.8858	0.027*	
C6	0.4278 (4)	0.8846 (3)	0.69071 (8)	0.0204 (5)	
H6	0.5870	0.9191	0.6882	0.024*	
C7	0.2393 (4)	0.8574 (3)	0.64086 (8)	0.0196 (5)	
C8	−0.1198 (4)	0.7629 (3)	0.59024 (8)	0.0230 (5)	
H8	−0.2775	0.7109	0.5804	0.028*	
C9	0.0049 (4)	0.8353 (3)	0.55333 (8)	0.0240 (5)	
H9	−0.0522	0.8433	0.5137	0.029*	
C10	0.2326 (5)	0.8955 (3)	0.58482 (8)	0.0218 (5)	
H10	0.3571	0.9512	0.5705	0.026*	

### Atomic displacement parameters (Å^2^)

**Table:**

	*U*^11^	*U*^22^	*U*^33^	*U*^12^	*U*^13^	*U*^23^
O1W	0.0245 (9)	0.0239 (7)	0.0224 (7)	−0.0061 (6)	0.0065 (7)	0.0001 (6)
N1	0.0242 (10)	0.0196 (9)	0.0231 (9)	−0.0003 (8)	0.0066 (8)	0.0013 (7)
N2	0.0194 (10)	0.0269 (10)	0.0200 (8)	−0.0038 (8)	0.0060 (8)	0.0001 (7)
N3	0.0214 (9)	0.0194 (8)	0.0199 (8)	−0.0005 (7)	0.0027 (8)	0.0005 (7)
N4	0.0229 (10)	0.0191 (8)	0.0200 (8)	−0.0011 (8)	0.0062 (7)	0.0014 (7)
C1	0.0202 (11)	0.0159 (9)	0.0217 (10)	0.0028 (9)	0.0048 (9)	0.0013 (8)
C2	0.0269 (12)	0.0195 (9)	0.0278 (10)	0.0027 (9)	0.0120 (10)	0.0032 (9)
C3	0.0361 (13)	0.0211 (10)	0.0215 (9)	0.0040 (10)	0.0102 (10)	0.0029 (9)
C4	0.0335 (13)	0.0191 (10)	0.0198 (10)	0.0034 (10)	0.0007 (10)	−0.0001 (9)
C5	0.0251 (13)	0.0178 (9)	0.0241 (10)	0.0005 (9)	0.0025 (10)	0.0003 (8)
C6	0.0209 (11)	0.0176 (10)	0.0241 (9)	−0.0001 (8)	0.0080 (10)	−0.0016 (8)
C7	0.0221 (12)	0.0166 (9)	0.0210 (10)	0.0006 (9)	0.0069 (9)	0.0005 (8)
C8	0.0226 (12)	0.0209 (10)	0.0243 (9)	−0.0008 (9)	0.0027 (9)	−0.0020 (8)
C9	0.0301 (13)	0.0219 (10)	0.0179 (9)	0.0025 (9)	0.0013 (9)	0.0005 (9)
C10	0.0243 (12)	0.0188 (10)	0.0234 (10)	−0.0005 (9)	0.0080 (9)	−0.0008 (8)

### Geometric parameters (Å, °)

**Table:**

O1W—H1W	0.842 (10)	C3—C4	1.392 (4)
O1W—H2W	0.840 (10)	C3—H3	0.9500
N1—C1	1.340 (3)	C4—C5	1.375 (3)
N1—C2	1.350 (3)	C4—H4	0.9500
N2—N3	1.364 (3)	C5—H5	0.9500
N2—C1	1.382 (3)	C6—C7	1.435 (3)
N2—H2N	0.886 (10)	C6—H6	0.9500
N3—C6	1.289 (3)	C7—C10	1.390 (3)
N4—C8	1.357 (3)	C8—C9	1.375 (3)
N4—C7	1.380 (3)	C8—H8	0.9500
N4—H4N	0.879 (10)	C9—C10	1.411 (3)
C1—C5	1.396 (3)	C9—H9	0.9500
C2—C3	1.377 (3)	C10—H10	0.9500
C2—H2	0.9500		
			
H1W—O1W—H2W	107 (2)	C3—C4—H4	119.8
C1—N1—C2	117.39 (19)	C4—C5—C1	118.3 (2)
N3—N2—C1	118.06 (17)	C4—C5—H5	120.9
N3—N2—H2N	119.3 (16)	C1—C5—H5	120.9
C1—N2—H2N	121.9 (16)	N3—C6—C7	118.4 (2)
C6—N3—N2	119.13 (18)	N3—C6—H6	120.8
C8—N4—C7	109.25 (17)	C7—C6—H6	120.8
C8—N4—H4N	127.9 (16)	N4—C7—C10	107.7 (2)
C7—N4—H4N	121.4 (17)	N4—C7—C6	121.43 (19)
N1—C1—N2	118.01 (18)	C10—C7—C6	130.8 (2)
N1—C1—C5	122.68 (19)	N4—C8—C9	108.4 (2)
N2—C1—C5	119.31 (19)	N4—C8—H8	125.8
N1—C2—C3	124.2 (2)	C9—C8—H8	125.8
N1—C2—H2	117.9	C8—C9—C10	107.86 (18)
C3—C2—H2	117.9	C8—C9—H9	126.1
C2—C3—C4	117.1 (2)	C10—C9—H9	126.1
C2—C3—H3	121.4	C7—C10—C9	106.7 (2)
C4—C3—H3	121.4	C7—C10—H10	126.6
C5—C4—C3	120.3 (2)	C9—C10—H10	126.6
C5—C4—H4	119.8		
			
C1—N2—N3—C6	−178.42 (18)	N2—N3—C6—C7	179.22 (17)
C2—N1—C1—N2	179.37 (18)	C8—N4—C7—C10	1.2 (2)
C2—N1—C1—C5	0.2 (3)	C8—N4—C7—C6	−177.71 (19)
N3—N2—C1—N1	15.4 (3)	N3—C6—C7—N4	−11.2 (3)
N3—N2—C1—C5	−165.42 (17)	N3—C6—C7—C10	170.2 (2)
C1—N1—C2—C3	0.0 (3)	C7—N4—C8—C9	−1.2 (2)
N1—C2—C3—C4	0.2 (3)	N4—C8—C9—C10	0.8 (2)
C2—C3—C4—C5	−0.6 (3)	N4—C7—C10—C9	−0.6 (2)
C3—C4—C5—C1	0.8 (3)	C6—C7—C10—C9	178.1 (2)
N1—C1—C5—C4	−0.6 (3)	C8—C9—C10—C7	−0.1 (2)
N2—C1—C5—C4	−179.75 (18)		

### Hydrogen-bond geometry (Å, °)

**Table:**

*D*—H···*A*	*D*—H	H···*A*	*D*···*A*	*D*—H···*A*
O1w—H1w···N1	0.842 (10)	2.037 (11)	2.870 (2)	170 (3)
O1w—H2w···N3^i^	0.840 (10)	2.078 (12)	2.899 (3)	166 (3)
N2—H2n···O1w^ii^	0.886 (10)	2.092 (12)	2.959 (3)	166 (2)
N4—H4n···O1w	0.879 (10)	1.971 (11)	2.831 (2)	165 (2)

Symmetry codes: (i) −*x*, *y*−1/2, −*z*+3/2; (ii) −*x*+1, *y*+1/2, −*z*+3/2.
